# Water Sorption in Hybrid Polyester/Glass/Jute Composites Processed via Compression Molding and Vacuum-Assisted Resin Transfer Molding

**DOI:** 10.3390/polym15224438

**Published:** 2023-11-16

**Authors:** Rudá Aranha, Mario A. Albuquerque Filho, Cícero de Lima Santos, Viviane M. Fonseca, José L. V. Rivera, Antonio G. B. de Lima, Wanderley F. de Amorim, Laura H. Carvalho

**Affiliations:** 1 Escuela de Ingeniería Mecánica, Pontifícia Universidad Católica de Valparaíso 1, Valparaíso 2340025, Chile; jose.valin@pucv.cl; 2 Post-Graduate Program in Materials Science and Engineering, Federal University of Campina Grande, Campina Grande 58429-900, Brazil; mario_alberto1910@hotmail.com; 3 Mechanical Engineering Department, Federal University of Campina Grande, Campina Grande 58429-900, Brazil; cicero.santos@ufcg.edu.br (C.d.L.S.); antonio.gilson@ufcg.edu.br (A.G.B.d.L.); engenhariabrasileira1@gmail.com (W.F.d.A.J.); 4 Textil Engineering Department, Federal University of Rio Grande do Norte, Natal 59078-970, Brazil; fonseca.vmf@gmail.com

**Keywords:** hybrid composites, jute fiber, VARTM, water sorption

## Abstract

The aim of this work is to analyze water sorption in hybrid polyester/glass fabric/jute fabric composites molded via compression and VARTM (Vacuum-Assisted Resin Transfer Molding). The laminates were produced with five different stacking sequences and subjected to water sorption testing at room temperature, 50 °C and 70 °C. This study consisted of two stages: experimental and theoretical stages. The composites had a fiber volume content ranging from 30% to 40%. Water absorption and diffusion coefficient in the hybrid composites were intermediate to those reinforced with a single type of fiber. There were no significant differences in these properties based on fiber arrangement once the composites reached saturation. Diffusion coefficient values were higher for specimens with jute fiber on at least one of the outer surfaces. Water sorption rates increased with higher immersion temperatures. The water sorption at saturation point was not affected by the manufacturing process. Among the hybrid composites, those with jute on the surfaces showed the highest diffusion coefficient, while those with glass on the surface had the lowest values. Higher diffusion coefficient values were observed at temperatures of 50 °C and 70 °C. The main influencing factors on the absorbed moisture content for composites are the presence and content of jute fibers in the system and the immersion temperature. The manufacturing process does not affect the water sorption at saturation point.

## 1. Introduction

GFRP composites are increasingly being used as an alternative to traditional materials due to their low density, high strength, and stiffness. Aiming to reduce the environmental impact and preserve the environment, vegetable fibers are replacing synthetic fibers as reinforcement in composites, even if the mechanical properties of vegetable fibers are lower than synthetic ones. A solution to this problem is the partial substitution of synthetic fibers by plant fibers to create a hybrid composite [1]. Jute fibers have been used for this purpose because they are abundant, available in many countries, have low financial cost, and a good set of mechanical properties [2].

Fibrous hybrid composites are materials in which two or more different fibers—synthetic or natural—are used to reinforce a given matrix. Fibrous hybrid composites have intermediate mechanical properties between those of the same composite reinforced by each one of the fibers [3]. Therefore, hybrid composites reinforced by plant fibers or plant/synthetic hybrids cannot be intended for high-performance and costly applications because vegetable fibers have lower mechanical properties than synthetic fibers [1]. Even so, the use of these hybrid composites allows the material to reach suitable properties and meet project requirements, as well as improving a specific property of the material through the addition of a second reinforcement [4,5,6].

In fibrous hybrid composites, the fibers can be arranged in several different ways: interlaminar, intralaminar, and mixed [7,8,9,10]. Some other factors have an influence on the properties of hybrid composites: the mechanical properties and nature of the fibers, the length of the different types of fibers, and the quality of the interfacial connection between the different fibers and the matrix [10]. The synergistic effect is also reported for hybrid composites with two or more resin systems or additional constituents such as inserts, nanoparticles, and additives [11,12,13]. The fiber stacking sequence is another parameter that directly influences the mechanical properties of hybrid composites [14]. Some authors investigated the effects of adding glass fiber in composites reinforced with natural fibers [15,16,17,18,19,20,21]. All of them concluded that the addition of glass fibers increased the mechanical properties of these composites.

The properties of materials change over time, suffering degradation due to external effects. Most polymeric composites are sensitive to light, humidity, and heat, among other aggressive environments. The exposure of a material to aggressive environments generates changes over time that can be observed in engineering properties such as strength and rigidity; physical characteristics such as density; or in chemical characteristics such as reactivity to chemical products [22]. The degradation of composites can occur not only with the degradation of the material components, but also with the loss of interaction between them, that is, the deterioration of the fiber/matrix interface [15]. Glass fibers experience significant mechanical property losses due to corrosion mechanisms promoted by environmental exposure [23].

Aging tests are used to investigate the degradation of composites under service conditions. Accelerated or natural aging tests can be performed to produce physical and/or chemical degradation. For instance, in the water sorption test, the material is immersed in an aqueous solution, with or without controlled temperature, in order to degrade at an accelerated rate. This is a way of understanding the material’s behavior when exposed to humidity. The glass transition temperature of thermosetting systems is modified when they are immersed in water at different temperatures until the saturation point is reached [24].

One-way composite materials absorb moisture into their structure through voids. The voids in the structure of the material will compromise the mechanical properties not only because of their existence, but also because they increase moisture absorption. Water absorption by composites does not occur only due to the existence of voids, but also due to the chemical affinity and type of matrix, temperature, polarity, diffusivity, and hydrogen bond formation, in addition to the nature, volumetric fraction, orientation, porosity, and geometry of fibers or fabrics [25,26,27].

Sorption is the phenomenon of mass transfer, where molecules from a liquid phase are associated with an immobile phase [28]. Water sorption in the composites followed a Fickian behavior [27,29]. Water sorption can be divided into adsorption that is related to the solid/fluid interface, where there is an accumulation of water molecules on the solid surface of the material, and absorption that occurs when water molecules penetrate the solid surface and settle inside the material. Water sorption in polymeric composites can be quite complex. When considering temperature variation, hygrothermal aging mainly damages the matrix, such as partial or total swelling and the formation of microcracks. This initial damage induces other damage mechanisms, such as interfacial debonding, delamination, fractures along the interface, resin particle loss, and fiber rupture [27,30,31,32,33,34,35,36]. Matrix swelling caused by water absorption is generally harmful to the fiber/matrix interface due to fiber detachment, which reduces the mechanical performance of the composite [27,32,37,38]. The diffusion of water molecules into polymeric networks will act as plasticizers. Plasticization tends to modify the glass transition temperature and decrease the strength of the composite [39]. On the other hand, moderate plasticization can also improve fracture toughness, preventing crack propagation in the material [40]. With a better adhesion between the reinforcement and the matrix, there was a reduction in water absorption, the diffusion coefficient, and swelling of the samples [29]. The fiber stacking sequence in hybrid composites should also be observed since it influences the water absorption of the composites [14,41,42].

When plant fibers are used as a reinforcement in polymer composites, the effects of water sorption on the composite are even more severe. Plant fibers, which are hydrophilic, are incompatible with thermoset resins, which are hydrophobic [31,32,43]. Therefore, the binding force becomes an impressive property to improve the adhesion between the fiber and the matrix. A decrease in the mechanical properties is expected due to the hygroscopic nature of these fibers and the poor fiber/matrix interface. Under constant environmental conditions, moisture content tends to increase with the volumetric fraction of plant fibers in the composite [32]. Such property losses were observed for polymeric composites reinforced by plant fibers of different nature, such as sisal [44], bamboo [45], flax [27,46], wood [47], hemp [33], and jute [48].

With the use of composite materials in various industries, understanding their behavior in real situations is essential. Therefore, correlating environmental effects such as temperature and humidity variations is important. However, due to the time and cost required for experimental studies in the actual conditions of material use, it is common to use different accelerated aging techniques as a more feasible alternative. The moisture content of fiber-reinforced polymer matrix composites has been extensively discussed in the literature [36,38,40,49,50,51,52,53,54,55,56,57,58]. However, investigations into the water sorption of hybrid composites reinforced with vegetable and synthetic fibers, such as the polyester/glass/jute composites, with different fiber stacking configurations on material properties, are still needed.

This study aims to investigate the effects of water sorption on polyester/fiberglass/jute fiber composites. The composites were processed using two methods: compression and Vacuum-Assisted Resin Transfer Molding (VARTM), and the laminates had five fiber stacking sequences. Water sorption tests were conducted, and it was possible to determine the water diffusion coefficient for these composites at different temperatures using the one-dimensional equation of Fick’s second law for a flat plate.

After the tests, it was concluded that the jute fiber composites showed the highest moisture absorption and estimated diffusion coefficient at saturation point. The fiberglass composites exhibited the lowest absorption content, while the hybrid composites had an intermediate absorption rate. Higher water temperatures during the tests increased the moisture absorption rate for all composites. The hybridization of jute fibers with glass fibers reduced the amount of water absorbed by the composites compared to jute fiber composites. It was observed that the highest rate of water absorption by the composites occurred within the first 50 h of immersion. Regardless of the water sorption test condition, at saturation point, differences in the results were not observed when comparing the processing methods. The main influencing factors observed on the absorbed moisture content are the presence and content of jute fibers in the system and the processing method used in composite manufacturing. The novelty of this work is based on the following:

- The literature does not address the combinations and analyses used here when comparing hybrid composites reinforced with vegetable and synthetic fibers after immersion in water at different temperatures with different stacking sequences and manufactured using two different methodologies.
- This study allows a deeper understanding of the experimental water sorption behavior of this composite material under various working conditions to be obtained.

## 2. Materials and Methods

### 2.1. Materials

In this work, orthophtalic unsaturated polyester resin 10,316 produced for Reichhold, from Mogi das Cruzes, Brazil (Table 1), catalyzed with MEKP BUTANOX M-50 supplied by IBEX Chemical, Recife, Brazil, was used. The reinforcements used were type E glass fiber fabrics with a gramature of 330 g/m² as supplied by Redelease ltd, located in Sorocaba, Brazil, and jute plain weave fabric with a gramature of 330 g/m², manufactured by Cia. Têxtil, located in Castanhal, Brazil.

### 2.2. Manufacture of Composites

**Figure 1:**
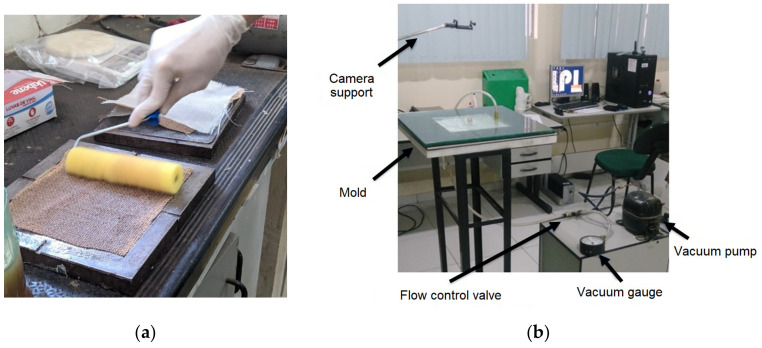
Manufacture of composite plates: (**a**) compression molding; (**b**) VARTM.

The composites investigated here were manufactured using two different methods: compression molding (Figure 1a) and VARTM (Figure 1b).

Regardless of the manufacturing method, each composite has four layers of glass fabric (G) and/or jute plain weave fabric (J) as the reinforcement. Hybrid composites were manufactured with two layers of each type of fabric arranged in different stacking sequences. The compression-molded composites were manufactured with the following layer stacking sequence: GGGG; JJJJ; GJGJ; JGGJ; GJJG. The composites manufactured by VARTM were manufactured with two stacking sequences: GJJG and JGGJ. Therefore, the effect of the fabrication method (compression or VARTM) can be compared between GJJG-C and GJJG-R composites, where ‘C’ stands for compression molding and ‘R’ stands for the VARTM process, and where ‘C’ stands for compression molding and ‘R’ stands for the VARTM process.

Compression molding of the plates was carried out in a metallic mold measuring 200 mm × 180 mm. The fabric layers were manually placed in the mold, laminated with a foam roller, and compressed at room temperature with 9 Ton in a Marconi uniaxial hydraulic press for 24 h before demolding and post-curing in an air circulation oven at 60 °C. VARTM processing was carried out in a rectangular mold with the same dimensions (200 mm × 180 mm), using a glass base and vacuum bag (Figure 1b), straight flow front, two entry points, and one exit point for resin with a diameter ¼’’ and a vacuum pressure of −0.3 bar.

At the end of the processing, the plates were weighed, and the volumetric fractions of the fibers could be determined. For this, the theoretical method (Equation (1)) was used [14].

(1) Vf=wjρj+wgρgwjρj+wgρg+wmρm,

where *V_f_* is the volumetric fraction of the fibers of the laminate; *w_j_*, *w_g_,* and *w_m_* are the masses of the jute fibers, glass fibers, and matrix, respectively; and *ρ_j_*, *ρ_g_,* and *ρ_m_* are the densities of the jute fiber, glass fiber, and matrix, respectively.

The compression-molded and VARTM plates were cut on a CNC mill. The specimens obtained had dimensions of 20 mm × 20 mm for the water sorption tests according to the ASTM D570—81 Standard [59].

### 2.3. Water Sorption Test—Experimental

The effects of water sorption by the material were evaluated through the mass variation as a function of the immersion time of the composites. The samples submitted to the water sorption test had dimensions of 20 mm × 20 mm, as suggested by the ASTM D570—81 Standard [59]. The samples were sealed at the edges with resin as a way to prevent the fibers from having direct contact with water and absorbing by capillarity. The samples were dried in a vacuum oven at 60 °C for 4 h to reach a constant weight, ensuring that no residual moisture was present, before being placed in an aqueous medium. The specimens were weighed before and after the water absorption test at room temperature, T = 50 °C and T = 70 °C for up to 30 days (or until saturation). The water absorption curves for each type of composite as a function of immersion time were then plotted.

Equation (2) shows how to obtain water absorption as a function of time (*M_t_*).

(2) $$M_{t}=\frac{W_{t}-W_{0}}{W_{0}}\;100\left(\%\right),$$

where *W_t_* is the mass of wet samples at time t and *W*_0_ is the initial mass of dry samples.

### 2.4. Water Sorption Test—Theoretical

For the study of theoretical water sorption, a simplified Fickian diffusional model was used. It was assumed that the samples are flat plates, and the humidity flux is transient one-dimensional. Water sorption in transient regime is described by Fick’s 2nd law (Equation (3)) [60].

(3) $$\frac{\partial_{c}}{\partial_{t}}=D\frac{\partial_{c}^{2}}{\partial x^{2}},$$

Making the necessary adjustments and considering that *M_t_* is the total amount of diffusing substance that entered the plate at time *t*, and *M*_∞_ is the total amount of diffusing substance after infinite time, we have Equation (4).

(4) MtM∞=2Dtl212π−12,

In the initial stage of absorption, water absorption in time *t* (*M*_*t*_) increases linearly with √*t* and *M*_∞_, which are associated with mass gain when the material approaches the saturation point. If we consider that the water absorption behavior follows the Fickian diffusion pattern, it can be described by Equation (5) [23,26,33,60,61,62,63,64].

(5) $$\frac{M_{t}}{M_{\infty }}=4\sqrt{\frac{Dt}{\pi h^{2}}},$$

where h is the sample thickness. The average diffusion coefficient (*D*) is determined by the water absorption capacity (*M*_∞_) and the kinetic constant of water absorption (*k*), obtained by the slope of the graph *M_t_* versus *t*^1/2^, described by Equation (6).

(6) $$D=\pi {\left(\frac{kh}{4M_{\infty }}\right)}^{2},$$

## 3. Results and Discussion

### 3.1. Volumetric Fraction of Fibers

Equation (1) was used to determine the fiber weight fraction and fiber volume fraction of the composites. The density of the fibers used for the theoretical calculation were 1.5 g/cm^3^ (jute) and 2.54 g/cm^3^ (glass). The values obtained for total and relative fiber weight and volume fractions are shown in Table 2.

Glass fabric composites showed the highest values of fiber volume fraction, with an average of approximately 39%. The jute fabric composites showed an average value of 36% of the fiber volumetric fraction. The fiber volume fraction of hybrid composites was lower for composites manufactured using compression molding (30%) when compared with composites manufactured using VARTM (37%). These results were expected because the VARTM provides better compaction of the fibers during manufacturing compared to the compression molding method due to the use of a vacuum. The applied vacuum together with the resin flow front during manufacturing promotes more efficient air removal, thus reducing the void content in the composites.

### 3.2. Water Sorption: Experimental Analysis

**Figure 2:**
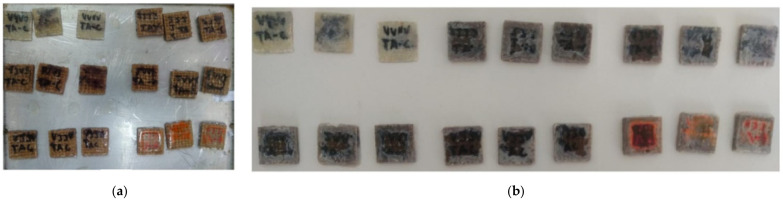
Water sorption test of the composites: (**a**) before the test; (**b**) at the end of the test (696 h).

Figure 2 shows the composites samples before and after the water sorption test at room temperature.

Surface changes were observed in the samples due to water absorption. The samples showed a change in color with a more “whitish” tone. Swelling of the samples at the end of the test caused by water absorption was also observed. Similar results were reported by [49].

**Figure 3:**
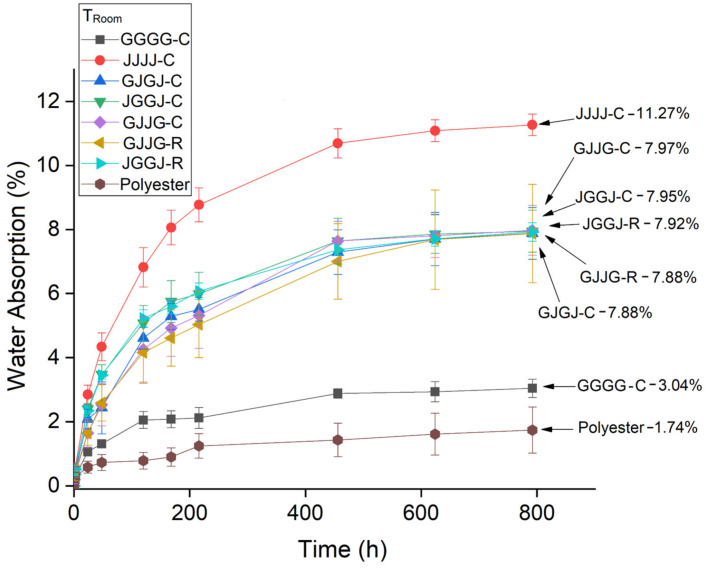
Water absorption at room temperature by the composites as a function of time (696 h).

**Figure 4:**
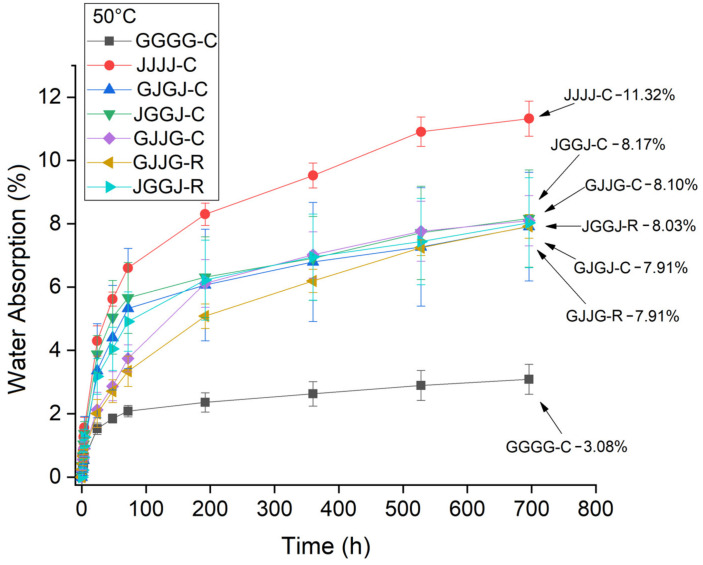
Water absorption at T = 50 °C by the composites as a function of time (696 h).

**Figure 5:**
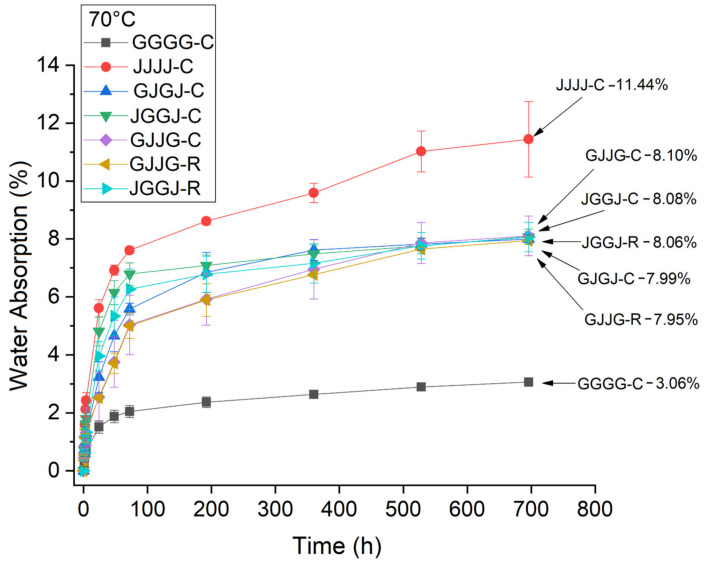
Water absorption at T = 70 °C by the composites as a function of time (696 h).

Water absorption as a function of time was measured according to Equation (2). Figure 3 shows the water absorption of the composites at room temperature, Figure 4 shows the water absorption at T = 50 °C, and Figure 5 shows water absorption at T = 70 °C. The exposure time of the samples for all conditions was 696 h.

It is clear that, for all exposure conditions (Figure 3, Figure 4 and Figure 5), the jute fabric-reinforced composite showed higher water absorption, while the glass fabric composites showed the lowest water absorption. This result was expected considering the hydrophilic nature of jute.

The polyester samples exposed to water at room temperature exhibited an absorption of 1.74%, which was the lowest value observed. Despite being composed of two synthetic materials, the glass fabric composites (polyester/glass fiber) showed higher water absorption than the polyester samples. This difference in absorption results between the glass fabric composite and the polyester samples occurred due to the manufacturing process of the composites. The wetting process of the glass fibers by the resin is not perfect, resulting in difficulty in filling, mainly for the micropores [65]. This difficulty in resin wetting implies the presence of small voids in the laminate, and these voids are responsible for the penetration of moisture into the composites.

The hybrid composites exhibited intermediate absorption rates for all studied conditions. The absorption values observed in the hybrid composites (Figure 3, Figure 4 and Figure 5) are closer to the values observed in the jute fabric composites. Due to its hydrophilic nature, jute is the main contributor to the total water absorption in hybrid systems. A slight variation in water absorption was found due to variations in the fiber stacking sequence and the method of manufacturing the hybrid composites (compression molding or VARTM). It should also be taken into account that the manufactured composites had a higher volumetric fraction of jute fiber than glass fiber, which corroborates these results.

The hybridization of jute fibers with glass fibers reduced the amount of water absorbed by the composites compared to jute fiber composites. The results from Figure 3, Figure 4 and Figure 5 indicate higher initial absorption in the hybrid composites with jute in the outer layers. Due to the hygroscopic nature and higher porosity of jute fibers, water diffuses through the resin and is absorbed in larger quantities when it comes into contact with these fibers. These results are consistent with the findings reported by others [9,66,67].

On the other hand, between 250 h and 350 h of immersion at room temperature, it was observed that the VJJV-C composites began to absorb more moisture among the hybrids and became the composite with the highest water absorption content. It is possible that within this time range (250–350 h), water particles reached the interior of the samples, as jute fibers absorbed more moisture than fiberglass. This behavior was not observed in the VJJV-R samples, which, although they showed an increase in water absorption, did not reach the same level as the samples manufactured via compression for the same immersion time. A higher rate of absorption for the VJJV-R samples occurred between 400 h and 600 h compared to the other hybrid composites. We can argue that this delay in the comparison between the samples is due to the fact that moisture has more difficulty penetrating the VARTM-manufactured samples. In longer immersion times (above 600 h), the small differences in water absorption between the hybrid composites were no longer evident.

For the conditions of T = 50 °C and T = 70 °C, the VJJV-C hybrid composites started to absorb more moisture than the other hybrids. However, this phenomenon was observed between 150 h and 200 h of immersion. We can consider that this acceleration in water absorption occurred due to the higher temperatures during the test. This behavior was not observed in the VJJV-R hybrid composite. Since there was a higher water absorption rate, after 500 h of testing, differences in absorption between the hybrid composites were not observed for these test conditions.

**Figure 6:**
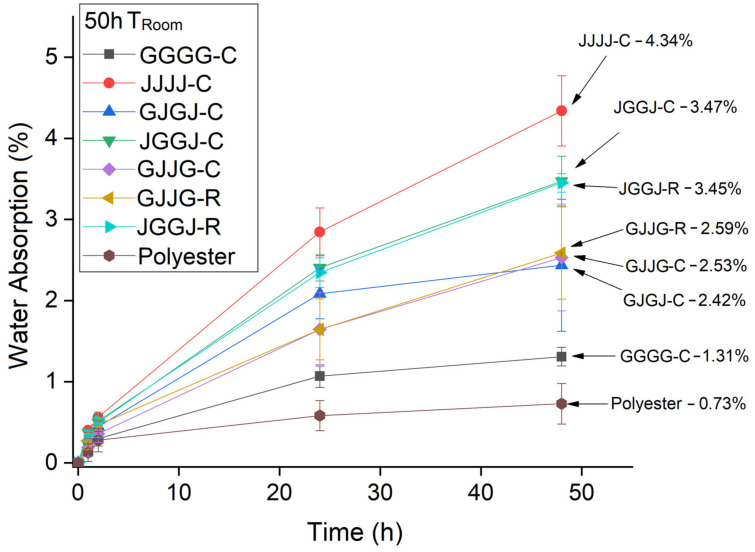
Absorption of water at room temperature by the composites in a time of 50 h.

**Figure 7:**
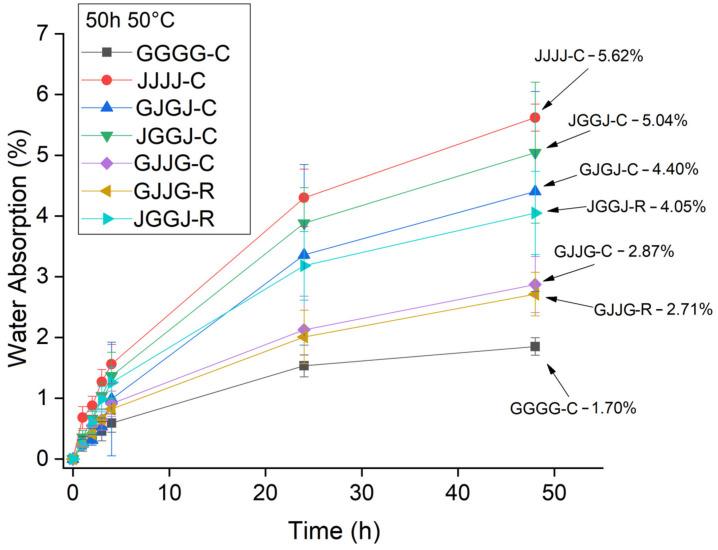
Absorption of water at T = 50 °C by the composites in a time of 50 h.

**Figure 8:**
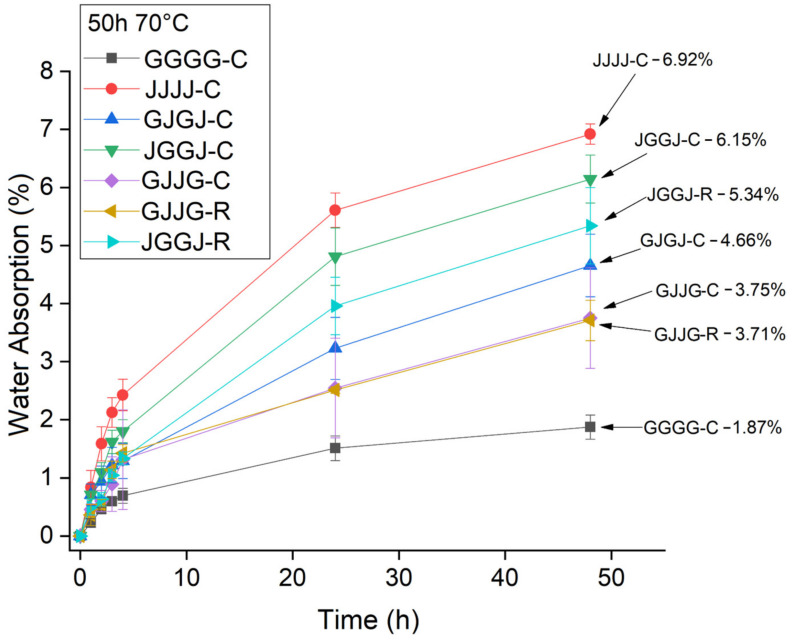
Absorption of water at T = 70 °C by the composites in a time of 50 h.

In Figure 6, Figure 7 and Figure 8, it was observed that the highest rate of water absorption by the composites occurred within the first 50 h of immersion. The JVVJ-C hybrid composites exhibited values close to those of the jute fiber samples. Regardless of the manufacturing method, the JVVJ composites absorbed more moisture than the VJJV composites. This result was expected due to the stacking sequence of these composites, with jute fibers on the surfaces.

The VJVJ-C samples absorbed more moisture than the VJJV-C and VJJV-R samples in the initial time period. This occurred because the VJVJ-C composite had a jute layer on at least one side of the composite.

In Figure 7, an unexpected result was observed: the VJVJ-C composite showed a higher water absorption value than the JVVJ-R composite. Naturally, this result can be attributed to the different manufacturing methods used. However, other hypotheses were considered in addition to the manufacturing methods since this reason alone is not sufficient to justify such a result. The hypothesis is that there were micro-cracks in the matrix, causing an increased absorption rate due to moisture penetration through these areas. Water sorption and temperature act in two ways in composites: Firstly, through the plasticization of the macromolecular network of the resin, leading to irreversible property losses [23]. Secondly, through microcracks in the matrix and fiber/matrix debonding caused by matrix swelling and an increase in the amount of moisture absorption.

When comparing the effect of processing methods on the water sorption of hybrid composites at room temperature, the results showed equivalence: both the VJJV-C (7.97%) and VJJV-R (7.88%) composites, and the JVVJ-C (7.95%) and JVVJ-R (7.92%) composites, showed similar values to each other (Figure 6), even though the VARTM-fabricated composites (~37.50% total fiber content) had a higher total theoretical fiber volume fraction compared to the compression-fabricated composites (~30.75%) (Table 2). These results can be explained by the greater compaction in the VARTM laminate due to the application of vacuum during the resin infusion process. The use of vacuum allows for greater compaction of the system and generates a lower void content, reducing moisture absorption inside the panels. The results for temperatures T = 50 °C and T = 70 °C were similar to those observed at room temperature (Figure 7 and Figure 8).

For the observed composite structures, the main influencing factors on the absorbed moisture content are the presence and content of jute fibers in the system and the processing method used in composite manufacturing, with the VARTM-fabricated composites showing similar absorption values for a higher jute fiber content compared to the compression-fabricated composites.

**Figure 9:**
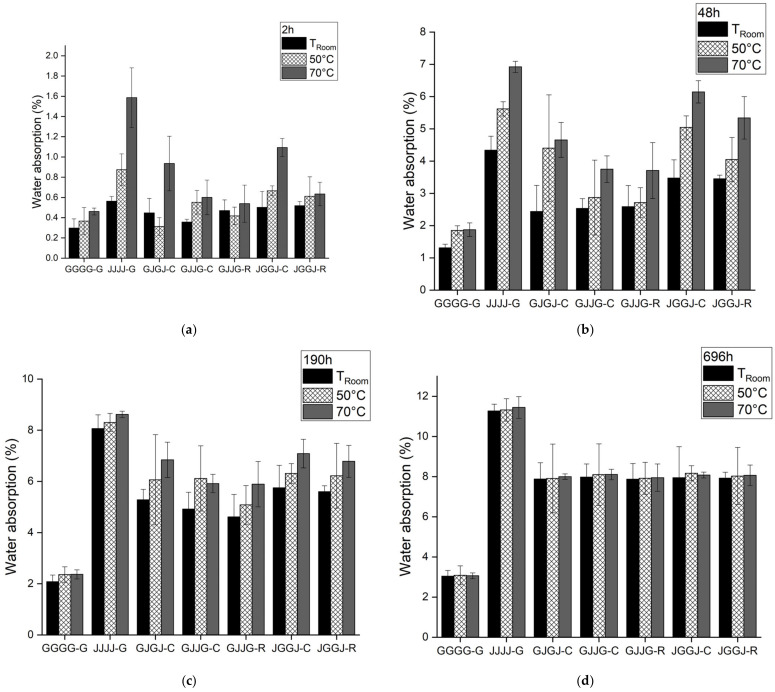
Comparison between sorption tests at room temperature, T = 50 °C and T = 70 °C for different immersion times: (**a**) 2 h; (**b**) 48 h; (**c**) 190 h; (**d**) 696 h.

When comparing the absorption rate of composites at room temperature, T = 50 °C and T = 70 °C, an analysis was conducted for different immersion times (Figure 9). It was observed that regardless of the immersion time, samples immersed at room temperature showed the lowest water absorption rates, samples immersed at 50 °C showed intermediate rates, and samples immersed at 70 °C showed the highest absorption rates. This result was expected because an increase in temperature promotes an increase in molecular movement, favoring the penetration of moisture into the material.

It is also important to consider that the temperature of 70 °C is close to the glass transition temperature of the polyester matrix. The exposure of the material over time at 70 °C tends to cause surface degradation in the resin. In Figure 9d, it was observed that after 696 h, the composites reached saturation, showing very similar water absorption values regardless of the immersion temperature.

### 3.3. Water Sorption: Theoretical Analysis

The results shown in Table 3 indicate that the fiberglass composites had the lowest values for the diffusion coefficient (D), jute fiber composites had the highest values, while hybrid composites showed intermediate values for water sorption at room temperature. Among the hybrid composites, the JVVJ stacking sequence exhibited the highest diffusion coefficient values, VJVJ had intermediate values, and VJJV had the lowest values.

These results for the hybrid composites indicate that the presence of fiberglass on the surface of the laminates initially delayed the diffusion of moisture into the material. On the other hand, the hybrid composite with alternating fibers (VJVJ-C) showed an intermediate diffusion value compared to the other hybrid types due to the presence of jute on only one surface of the laminate. Glass fiber, with its hydrophobic nature, when placed on the surface of the composite, acts as a protective barrier against the penetration of moisture from the interior, thus slowing down water absorption. No significant differences were observed between the manufacturing methods.

**Figure 10:**
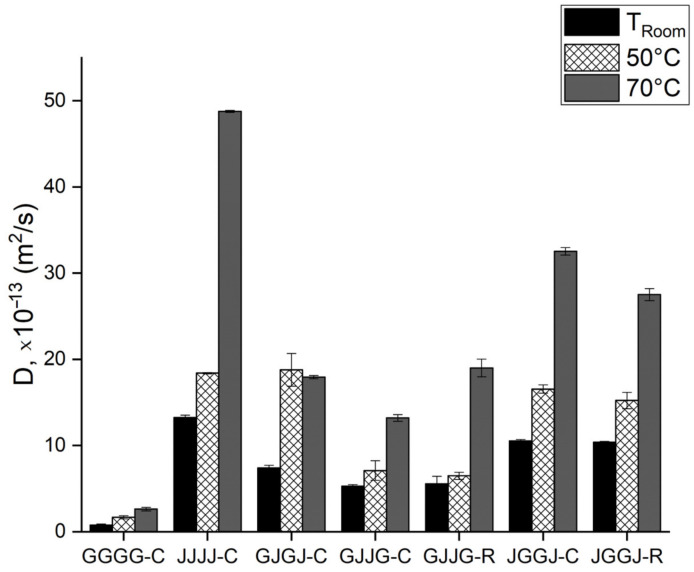
Comparative analysis of the diffusion coefficient of composites immersed in water at room temperature, 50 °C and 70 °C.

The diffusion coefficient of composites immersed at T = 50 °C and T = 70 °C (Table 4 and Table 5) showed higher values than composites immersed at room temperature. The comparison of these values for all composites is shown in Figure 10. These results are expected because an increase in temperature tends to increase molecular movement, thereby increasing the rate at which composites absorb moisture. When immersed at T = 70 °C, the composites exhibited a higher diffusion coefficient compared to composites immersed under different conditions. As previously mentioned, this is due to the fact that the temperature of T = 70 °C is close to the glass transition temperature of the polyester, leading to matrix degradation and contributing to greater moisture diffusion within the material.

Attention should be paid to Equations (3)–(6), which only consider the initial stage of water absorption. During this stage, water absorption increases linearly, and the kinetic absorption constant (k) is obtained from the slope of the Mt versus t1/2 graph, which is used to calculate the diffusion coefficient (D). Although the water absorption of hybrid composites shows similarities in saturation, differences in the absorption curve behavior were observed during the initial hours of exposure due to the stacking sequence of the laminate layers. Regardless of the temperature condition during immersion, composites with jute fiber on the surface of the laminate exhibited a higher diffusion coefficient compared to composites with fiberglass on the surface.

## 4. Conclusions

Water sorption in hybrid polyester/fiberglass/jute fiber composites was analyzed. It was possible to manufacture the composites using both methods: compression molding and VARTM.

- The composites showed theoretical fiber volume fraction values ranging from approximately 30% to 40%, with the VARTM-produced hybrid composites showing higher values than the compression-molded hybrid composites.
- During the water sorption tests, the composites reached saturation after 696 h. Among them, the jute fiber composites showed the highest moisture absorption content after 696 h. The fiberglass composites exhibited the lowest absorption content, while the hybrid composites had an intermediate absorption rate. Using higher water temperatures during the tests increased the moisture absorption rate for all composites. The hybridization of jute fibers with glass fibers reduced the amount of water absorbed by the composites compared to jute fiber composites.
- It was observed that the highest rate of water absorption by the composites occurred within the first 50 h of immersion. Regardless of the test condition, higher moisture absorption rates were observed in the hybrid composites with jute layers on at least one of the surfaces during the initial 50 h.
- At long times, above 600 h, such differences were no longer observed for the composites immersed at room temperature (500 h for the composites immersed at 50 °C and 70 °C). Regardless of the water sorption test condition, at the time of 696 h, differences in the results were not observed when comparing the processing methods, as this is the time associated with sample saturation.
- When comparing the effect of processing methods on the water sorption of hybrid composites for all temperatures, the results showed equivalence. VJJV-C and VJJV-R composites, and the JVVJ-C and JVVJ-R composites, showed similar values to each other. VARTM-fabricated composites showed similar absorption values for a higher jute fiber content compared to the compression-fabricated composites.
- The estimation of the diffusion coefficient calculated through Fick’s second law showed that the jute fiber composites exhibited the highest diffusion coefficient, the fiberglass composites showed the lowest values, and the hybrid composites presented intermediate values. The JVVJ composites had a higher diffusion coefficient among hybrids due to the presence of jute fiber on the surfaces. The VJJV composites had the lowest values for the diffusion coefficient. Higher values of the diffusion coefficient were observed at temperatures of 50 °C and 70 °C when compared to the composites immersed at room temperature.
- For the observed composite structures, the main influencing factors on the absorbed moisture content are the presence and content of jute fibers in the system, temperature increase, and the processing method used in composite manufacturing.

## Figures and Tables

**Table 1:** Characteristics of resin—Reichhold guideline.

Characteristics	Values
Viscosidade Brookfield viscosity at 25 °C sp3: 60 rpm (CP)	250–350
Thixotropy index	1.30–2.10
Solid content—Reichhold method (%)	55–63
Density at 25 °C (g/cm^3^)	1.07–1.11
Acidity index (mgKOH/g)	30 maximum
Exothermic Curve at 25 °C - Gel time (min) - Single interval (min) - Maximum temperature (°C)	
	5–7 8–14 140–180
Post-cure	60 °C

**Table 2:** Total and relative fiber weight and volume fraction of manufactured composites.

Composites	Glass Fiber Weight Fraction (%)	Jute Fiber Weight Fraction (%)	Total Fiber Weight Fraction of Composites (%)	Fiber Volume Fraction of Composites (%)	Total Fiber Volume Fraction of Jute (%)
GGGG-C ^1^	59.70 ± 3.27	0	59.70 ± 3.27	39.35 ± 3.21	0
JJJJ-C	0	44.10 ± 2.10	44.10 ± 2.10	36.87 ± 1.97	36.87 ± 1.97
GJGJ-C	15.23 ± 0.43	26.10 ± 0.74	41.33 ± 1.17	30.68 ± 1.02	24.77 ± 0.90
GJJG-C	15.23 ± 1.06	26.11 ± 1.82	41.34 ± 2.89	30.72 ± 2.55	24.81 ± 2.24
GJJG-R ^1^	17.77 ± 1.77	30.46 ± 3.04	48.23 ± 4.82	36.99 ± 4.48	30.42 ± 4.06
JGGJ-C	15.28 ± 1.16	26.19 ± 2.00	41.47 ± 3.16	30.83 ± 2.76	24.91 ± 2.42
JGGJ-R	18.15 ± 1.18	31.12 ± 2.02	49.28 ± 3.20	37.94 ± 3.00	31.27 ± 2.73

^1^ C and R indicate the manufacturing method: compression molding (C) and VARTM (R).

**Table 3:** Water absorption capacity (M_∞_), kinetic constant (k), and diffusion coefficient (D) for composites immersed in water at room temperature.

Composites	M_∞_ (%)	k (h^−1^)	D, ×10^−13^ (m^2^/s)
VVVV-C	3.04	0.00189	0.76
JJJJ-C	11.27	0.00639	13.26
VJVJ-C	7.88	0.00437	7.40
VJJV-C	7.97	0.00373	5.27
VJJV-R	7.88	0.00370	5.56
JVVJ-C	7.95	0.00514	10.54
JVVJ-R	7.92	0.00508	10.37

**Table 4:** Water absorption capacity (M_∞_), kinetic constant (k), and diffusion coefficient (D) for composites immersed in water at T = 50 °C.

Composites	M_∞_ (%)	k (h^−1^)	D, ×10^−13^ (m^2^/s)
VVVV-C	3.08	0.00284	1.67
JJJJ-C	11.32	0.00756	18.40
VJVJ-C	7.91	0.00699	18.78
VJJV-C	8.10	0.00440	7.10
VJJV-R	7.91	0.00401	6.48
JVVJ-C	8.17	0.00662	16.55
JVVJ-R	8.03	0.00624	15.22

**Table 5:** Water absorption capacity (M_∞_), kinetic constant (k), and diffusion coefficient (D) for composites immersed in water at T = 70 °C.

Composites	M_∞_ (%)	k (h^−1^)	D, ×10^−13^ (m^2^/s)
VVVV-C	3.06	0.00354	2.63
JJJJ-C	11.44	0.01244	48.77
VJVJ-C	7.99	0.0069	17.94
VJJV-C	8.10	0.00600	13.20
VJJV-R	7.95	0.00690	18.99
JVVJ-C	8.08	0.00918	32.54
JVVJ-R	8.06	0.00842	27.51

## Data Availability

Not applicable.
